# The relationship between shame, perfectionism and Anorexia Nervosa: A grounded theory study

**DOI:** 10.1111/papt.12425

**Published:** 2022-09-25

**Authors:** Tina L. M. Howard, Marc O. Williams, Debbie Woodward, John R. E. Fox

**Affiliations:** ^1^ Clinical Psychology, School of Psychology Cardiff University Cardiff UK; ^2^ Cardiff and Vale University Health Board Cardiff UK

**Keywords:** anorexia nervosa, Grounded theory, perfectionism, shame

## Abstract

**Objectives:**

The aim of this study was to explore the potential relationship between shame, perfectionism and Anorexia Nervosa (AN) and their impact on recovery from AN.

**Method:**

Semi‐structured interviews were conducted with 11 people currently accessing services for AN. Interviews were transcribed and analysed using constructivist‐grounded theory methodology.

**Results:**

A model was developed which found a vicious cycle between shame and perfectionism. Participants tried to alleviate their feelings of shame by striving for perfectionism, however failing caused them more shame.

Participants who disclosed childhood trauma believed their shame preceded their perfectionism. Participants who did not disclose trauma either believed their perfectionism preceded shame or they were unsure of which occurred first.

Participants' responses suggested the following pathways from perfectionism to AN: needing goals; the need for a perfect life including a perfect body and AN being something they could be perfect at. The pathways identified between shame and AN entailed mechanisms via which AN could be used to escape shame, either by seeking pride through AN, seeking to numb shame through AN, seeking to escape body shame and punishing the self. AN was found to feed back into shame in two ways: when people had AN they felt ashamed when they broke their dietary rules, and also simultaneously people felt ashamed of their AN as they were not able to recover.

Shame and perfectionism influenced one another in a cyclical pattern, in which shame drove perfectionism and not attaining high standards led to shame. Shame and perfectionism also impacted on recovery in several ways. AN functioned to numb participants' emotions, becoming part of their identity over time. AN also brought respite from a constant striving towards perfectionism. The need for a perfect recovery also influenced their motivation to engage in treatment, and fear of a return of strong emotions was another deterrent to recovery.

**Conclusion:**

The findings of this paper show perfectionism and shame to both be important in the aetiology and maintenance of AN and to have an impact on recovery from AN.

**Practitioner points**

- Shame and Perfectionism have been shown to be important in understanding eating disorders, including Anorexia Nervosa (AN). However, shame and perfectionism have not been considered together before, especially from a qualitative angle.
- Data showed that perfectionism and shame create a vicious cycle that drives both the eating disorder and low mood.
- It is important to consider how challenging perfectionism within clients is likely to evoke shame, which may lead to disengagement from therapy.

## BACKGROUND

Eating disorders (EDs) are characterised by significant eating disturbance and acutely poor body image. One type of ED is Anorexia Nervosa (AN), characterised by low body weight (BMI > 17.5) and restrictive eating, and may include episodes of bingeing and purging for some individuals (DSM‐V, 2013). Although AN is a relatively rare condition (Hudson et al., 2007), it is regarded as one of the most severe mental health difficulties and it has the highest mortality rate across all the mental health conditions (Arcelus et al., 2011; Fichter and Quadflieg 2016; Guinhut et al., 2021; Westmoreland et al., 2016). Treatments for AN often lack potency and, as a consequence, relapse rates are high. Berends et al. (2016) report that relapse rates can be as high as 57%, depending on the definition of relapse and length of follow‐up. These rates are in keeping with other research that has suggested that relapse will occur following treatment in approximately one third (35%–41%) of individuals with AN (Carter et al., 2004, 2012; McFarlane et al., 2008).

Blythin et al. (2018) highlight that most of the current models of EDs, including AN, tend to be cognitive (e.g. Waller & Kennerley, 2003) and although they have made significant contributions to treatments of EDs, they have often neglected emotional factors. Fox et al. (2012) argued that to increase the efficacy of treatment, emotions need to be central in both the theoretical models of ED and in their respective treatments. Self‐conscious emotions, such as shame and pride, have been shown to be important within eating disorders (e.g. Faija et al., 2017; Goss & Allan, 2009). Indeed, shame is higher within ED populations even when compared to other clinical groups and, as severity of symptoms increases, so does self‐reported levels of shame, especially body shame, within AN populations (Blythin et al., 2018; Skårderud, 2007). Previous research/theory has highlighted that shame consists of two main distinctions: external and internal shame. According to Gilbert (2010), internal shame involves negative self‐scrutiny and appraisal of self as flawed, worthless, unlikeable by others or weak, whilst within external shame, the self is viewed as flawed and unattractive by others. A further important distinction between globalised and focal shame was made by Skårderud (2007) who highlighted that shame can be very generalised (e.g. the self being perceived as being entirely flawed or worthless) or focal (e.g. focused on an aspect of the self that leads to shame without necessarily leading to globalised shame). For the purposes of this research study and building on the literature discussed above, shame has been defined as a complex, painful self‐conscious emotion that originates from a global self‐devaluation and perceived negative evaluations of the self by others.

Goss and Allan (2012) proposed a model where they formulated that shame and pride interact to drive the eating disorder. According to the model, shame leads to attempts to feel pride through restriction and weight loss or attempts to numb shame through bingeing. However, these strategies are unstable and lead to additional distress and shame, which further drives the ED. Not only can this resulting shame be both global and focal but, importantly, it can also be external (e.g. ‘others will think I am weak’) and internal (‘I am useless and have no control’). This model had further support from a grounded theory study by Faija et al. (2017), who highlighted how pride within AN can be initially ‘alluring’ but becomes ‘toxic’ over time and leads to further shame, restriction and distress.

Although research looking at self‐conscious emotions in EDs has started to gather pace, there is still a lack of nuance about how these emotions interlink with other features of EDs. One area that has consistently been shown to be important in EDs, especially AN, is perfectionism. Dahlenburg et al. (2019) meta‐analysis of the relationship between AN and perfectionism reported that people with a diagnosis of AN have significantly higher levels of perfectionism when compared to other clinical groups (g = .41). Although they did acknowledge that there was a risk of publication bias in the literature, it is likely that perfectionism is a crucial factor in understanding AN. With Goss and Allen's model, which builds on the compassionate focused therapies models (e.g. Goss & Gilbert, 2002), it is likely that perfectionism and pride/shame interact, but there has not been any research to look at whether there is a relationship and what the nature of this relationship might be.

### Aims of the study

Studies have consistently found both perfectionism and shame to be relevant in the development and maintenance of eating disorders, including AN. However, these constructs have always been considered separately when contemplating their role in AN. This study aims to explore the potential relationship between shame, perfection and AN using a Grounded Theory approach. It also aims to look at the impact of shame and perfectionism on recovery from AN, as it is envisaged that understanding their relationship is likely to help further develop evidence‐based treatments and improve their efficacy.

## METHOD

### Design

Grounded theory methodology (Charmaz, 2006) was chosen as it provides a level of analysis that goes beyond the descriptive by generating a theory of the nature of the relationship between shame and perfection within AN. A key feature of grounded theory is that allows a flexible approach to data collection and questioning to develop a clear and ground theoretical understanding of the subject matter. As will described below, data collection started with purposive sampling, but due to the limits around recruitment full theoretical sampling was not possible. However, the interview was adapted to allow a fuller exploration of themes as they were emerging from the data. This allowed for further understanding and refinement of theoretical categories throughout the analysis (e.g. via constant comparison and analysis shaping further data collection).

### Recruitment, participants and data collection

Purposive sampling was used to recruit women with AN and, in order to ensure diagnosis, participants were recruited from NHS eating disorder services. Informed consent to take part in the study was gained by the treating clinician and each participants signed an informed consent form that was collected by the lead researcher (TH). At the start of the interview, each participant's understanding of the research process was checked before the interview started. One local outpatient eating disorder service agreed to take part in this study and, unfortunately due to the lack of a local eating disorders inpatient facilities, it was not possible to recruit inpatients. A key part of recruitment was that the treating clinician approached potential participants on their caseload, and they asked if they would like to take part in the study. Unfortunately, clinicians did not record details of who they asked to take part in this study, so we are not able to report data on recruitment rates. However, all participants who expressed an interest in this study and spoke to the lead researcher (TH) about being interviewed did eventually take part in the study.

Inclusion criteria were as follows: (1) Females over the age of 18 years and currently accessing a community eating disorders service, (2) a formal diagnosis of AN and (3) sufficiently fluent in English to partake in the research interview. Diagnosis was confirmed by a lead clinician with extensive experience of diagnosing and treating AN. Participants also completed the Eating Disorder Examination questionnaire (EDE‐Q 6.0; Fairburn & Beglin, 2008) and a demographics questionnaire. The EDE‐Q is a well‐established and robust measure of eating disorder symptoms and behaviours. It focuses on eating disorder‐related thoughts and behaviours, as well as body image. It also provides an overall Global Score that provides a combined score indicating severity of the ED, and research has concluded that a score above four indicates caseness for an ED (Mond et al., 2004). Participants' BMI was collected from patient records at the eating disorder services, with consent. The study received full UK NHS ethical approval. Sample size was led by theoretical sufficiency (Charmaz, 2006; Dey, 1999). The authors agreed that sufficiency was broadly reached after 11 interviews and, in agreement with Faija et al. (2017), theoretical sufficiency was achieved by rigour within the analysis and the categories were detailed enough to develop a robust theory.

Eleven women participated in the study. As can be seen in Table 1, all participants met criteria for AN and reported to have met criteria for a number of years. They were all interviewed face‐to‐face, and they completed the demographic questionnaire and the EDE‐Q at the start of the interview. All interviews were audio recorded and transcribed verbatim. All identifying information was removed from the transcripts to ensure that they were confidential.

**TABLE 1:** Participant characteristics

Participant	Age	Age at onset of AN	Duration of AN	Marital status	BMI	EDE‐Q global score
1	25	23	2 years	Single	17	4.925
2	41	15	26 years	Married	16.5	5.5
3	37	12	25 years	Single	16.7	4.82
4	22	15	7 years	Single	17.5	4.65
5	30	11	19 years	Single	17.5	6
6	20	16	4 years	Single	16	4.15
7	24	20	4 years	Single	15.4	4.87
8	25	17	8 years	Single	15.6	4.91
9	22	15	7 years	Single	17.5	3.95
10	22	15	7 years	Single	16.6	5.15
11	31	19	12 years	Single	16.9	6

#### Interview schedule

The interview schedule was developed through discussion with the research team. In order to prevent undue influence from the 2nd, 3rd and 4th authors on the data collection and analysis, questions were kept broad and were evolved throughout the actual process of data collection. The interview topics were:

1. **High Standards/Perfectionism**
  Examples of prompts were: ‘Do you set high standards for yourself?’, ‘What does it take for you to fell as if you have succeeded at something?’
2. **Comparison to others**
  Examples of prompts were: ‘How do you think you compare to other people?’
3. **Shame**
  Examples of prompts were: ‘How do you define shame?’, ‘Can you think of a time you have felt ashamed?’
4. **Relationship between shame, perfectionism and eating disorder**
  Examples of prompts were: ‘Does feeling ashamed lead you to try and be perfect?’, ‘Does not achieving perfectionism lead you to feel ashamed?’

In line with grounded theory, the interview schedule was revised after 4 interviews, with more emphasis on childhood experiences and the origins of shame and perfectionism. Interviews tended to last approximately 1 h. Interviews were conducted within the eating disorder service base and were collected over the end of 2018 and the beginning of 2019.

### Data analysis

The data were analysed by the first author in line with constructivist grounded theory methods (Charmaz, 2006). The analysis was undertaken by the first author (TH) who was a 35‐year‐old white, British trainee clinical psychologist at the time of data collection and analysis. She does not have any personal experience of eating disorders and has not worked in specific eating disorders services. She has worked with adults who have experienced eating disorders as well as with people with a history of trauma within secondary care services in the United Kingdom.

The data were initially coded on a line‐by‐line basis in order to define what was happening in the data and to begin constructing ideas about what it means. This process was undertaken within a word document and with the additional use of ‘post‐It notes’ to aid memo development. The codes stayed as close to the data as possible. The codes cannot be assumed to capture an empirical reality but instead represent the author's understanding of what was happening. Attention was paid to actions and processes rather than themes and structure. Throughout the analysis, attention was paid to searching for variation in the studied processes. The next stage of analysis was focused coding which involved studying and assessing the initial codes and making comparisons between them. This process of comparison allowed the author to consider what promising tentative categories were emerging. Again, this process was influenced by how the author interpreted the meanings of the codes. During the coding process, the author kept memos about the data and the concepts that were emerging. These emerging categories were then developed into an explanatory framework. In order to ensure that the analysis was reliable, coded transcripts were checked by a second rater with expertise in qualitative methods. Further, a reflective journal was utilised to ensure that appropriate bracketing of ideas and knowledge was undertaken throughout the analysis.

## RESULTS

**FIGURE 1:**
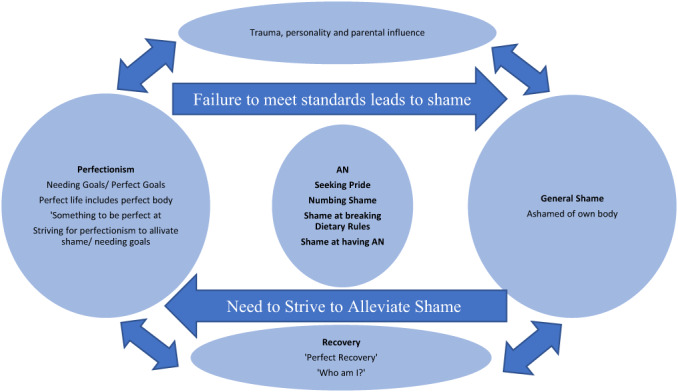
Model of the relationship between shame, perfectionism and AN [Colour figure can be viewed at wileyonlinelibrary.com]

An explanatory framework of the relationship between shame, pride and anorexia, and their effects on recovery was developed. Figure 1 provides a diagrammatic representation of the theory and the relationship between categories.

### Main findings

#### Over arching theme: Trauma, personality and parental influence leading to shame and perfectionism

Across the interviews, a number of early life factors were shared that suggested that childhood trauma and aversive parenting styles played a significant role in the development of shame and perfectionism for the participants. A number of participants spoke about quite severe trauma in their childhood and linked this to the origins of their shame. Crucially, within the analysis, this shame predated the emergence of perfectionism, which appeared to have the function of striving to allay the sense of judgement, either from others or the self. Indeed, one of the participants directly linked her perfectionism to the trauma of her father's physical and emotional abuse towards her and his punishing her for making mistakes. Another participant linked her perfectionism to being constantly told that she was not good enough by her mother:

When I do set myself standards that I think are good enough, there's always someone to say that it's not good enough, and usually that's my mother. Participant 10

Interestingly, a small number of participants did not disclose any trauma and believed that their perfectionism was an inherent personality trait. However, a closer examination of the data highlighted how these same participants also reported having critical or demanding parents and a good number were bullied at school. Within the analysis, the most parsimonious consideration of these divergences in the data, was one of insight.

#### Overarching theme: General shame

All participants reported experiencing high levels of shame. This shame touched all aspects of their lives, as they discussed feeling ashamed of who they were as people, feeling ‘second rate’ in comparison to other people and a general sense of feeling worthless. Given the high levels of criticism and aversive experiences growing up it is, perhaps, not surprising that there was such a strong sense of being worthless, ashamed and feeling ‘second rate’. This is summed up by participant 3:

I'm not as good as other people, I'm not as clever as other people, erm, I'm not as honest as other people Participant 3

Participants often felt globalised shame and were not able to identify anything positive about themselves.

I'm just ashamed to be me. Participant 2

As discussed above, this shame really drove the need to be ‘perfect’ as this was perceived as the only way to alleviate the overwhelming feelings of shame.

##### Ashamed of the body

Perhaps not surprisingly, many of the participants reported strong feelings of body shame that preceded their AN. Although not directly addressed within the data, a hypothesis emerged from the analysis about whether the global shame ‘found’ a focus upon the body, which may be behind the drive to lose weight and restrict their eating.

my body doesn't fit me, it's disgusting, I'm ashamed that I'm in this body and people have to witness it walking through town… I could just be thinking of my tummy area or something and then I have to restrict for days. Participant 2

The drive to lose weight and restrict their eating was intensified by perfectionism, which drives increasing pressure to lose weight. As will be discussed below, this then became linked to pride that further drives weight loss. Some participants expressed that they thought weight loss and having a better body would lead to social acceptance and to feeling better about themselves.

I always put on this expectation when I dieted for prom that at the end of it, when I got to prom I'd look amazing, and then other people would suddenly turn around and like me and accept me. And when prom wasn't how I wanted it to be, I just felt like, oh, maybe I need to keep going, maybe I didn't do it hard enough. Participant 8

#### Overarching theme: Perfectionism

Perfectionism took many forms for the participants. This perfectionism not only drove the sense of never being good enough but also directly linked to increasing levels of shame. So, for participant 11, it was not possible to undertake a task without it being perfect:

I would have to be in the perfectionist zone in order for me to carry out a task. Participant 11

But the nature of perfectionism often led to a strong sense of failure as powerfully highlighted by participant 5:

I'm not perfect enough to be a perfectionist. Participant 5

##### Perfect life includes perfect body

Participants spoke about pervasive perfectionism and wanting to be perfect in every area of life. This included wanting to have the perfect diet, exercise regime and also having a perfect body.

I feel like I always have to have the perfect body and if I don't eat I'll become more beautiful. Participant 10

People would strive towards what they thought was the perfect body and would restrict food intake and exercise to achieve perfection. Some participants were able to acknowledge that as the AN took hold, their perception of a perfect body became distorted. Striving for this perfect body helped relieve their sense of shame.

I think feeling ashamed of myself, or feeling embarrassed about myself, or feeling like I wasn't good enough, or wasn't worthy enough, it led me to developing a lot of habits, and to seek that perfection, that perfect lifestyle that all came together and you know I think that part of it was, the perfectionism in the diet and the way I looked. Participant 1

##### Something to be perfect at

As mentioned above, participants spoke about striving for perfectionism in all areas of life. They inevitably found that this always led to failure and shame as they were not able to be perfect. But one area where they felt they could be perfect was with their eating. Being perfect at AN seemed more obtainable than being perfect in other areas of life.

To be perfect you have to have it all, and you have to have a great job, and have a great social life, have this perfect exercise and diet regime and, I wanted to be perfect in all aspects. The only part of that I thought that I could control any part of was the diet and the exercise, so I felt that I had to get that perfect. Participant 1

Many participants spoke about wanting to have something they could control when other things in their life seemed out of their control.

I just couldn't control anything in my life at that moment, so that was the only thing I could control was what intake I was having. And yes, I thought life would be perfect if I lost weight. Participant 10

Being out of control for participants seemed to mean that things were imperfect. Either something unexpected had happened, or they were just struggling to maintain perfection across their life.

If I'm not happy with other areas like the house maybe, then I think that actually leads me to wanting to restrict more, because if I'm not doing well in that then at least I could be doing well in that. Participant 3

This increased their feelings of shame so striving to be perfect at AN helped to alleviate this shame.

##### Striving for perfectionism to alleviate shame

Participants expressed they would strive for perfectionism to alleviate their feelings of shame, and to try and feel good enough.

So, when I feel ashamed, I guess it pushes me to wanting to be good at something, so you just keep trying and trying… but it's never enough, you can always do better, I could never reach that perfection, I just couldn't get there. Participant 4

This sometimes came as a direct consequence of trauma, to avoid the negative repercussions of imperfections. Striving for perfectionism was also used as an attempt to suppress the criticism and demands from parents and to try and be good enough for their approval.

Being in that perfectionist zone keeps me away from the shame. And as long as I can keep in there, I'm not feeling that shame. Participant 2

##### Needing goals

Participants spoke about always needing goals and something to work towards to compensate for their shame. They felt they had to be continually striving to better themselves and to reach new targets. In contrast to the above where participants described striving towards AN when life became overwhelming, to avoid or counteract things in their life that they could not control, participants also described AN becoming dominant when they had no other goals and nothing else to focus on, and perhaps nothing else to relieve their shame.

when I found myself in this job, which wasn't very fulfilling, I took that drive for progress or perfection, you know, I wanted to have a project to work on so I made it my running… you know find fulfilment or feel as if I was doing something. Participant 1

#### Overarching theme: Failing to meet self‐imposed standards leads to shame

Participants were all aware that perfectionism was unachievable and eventually they would make a mistake or do something less than perfectly. This led to intense feelings of shame.

if I am a perfectionist about something and I fall short of it I automatically feel ashamed. Participant 1

Participants expressed shame at other people noticing their imperfections or mistakes that they made. They also expressed internal shame and said that failing to meet self‐imposed standards confirmed their negative sense of self.

The link between perfection and shame was described as two‐way process:

If I'm not perfect I feel ashamed and if I feel ashamed then I want to be perfect. Participant 1

Many described this as a loop that was continuously repeating, so when they felt shame at failed perfectionism they would strive even harder for that perfectionism to alleviate their feelings of shame, this again would inevitably fail and they would feel even more shame.

Participants also expressed that even when they did meet their self‐imposed standards they would continue to increase them.

Every time I got something right, it'd be like, oh okay, obviously I didn't set my standards high enough, let's go one higher. And then I'd just keep doing that until eventually it's too high, and there's no way you can get there. Participant 9

Even when goals were met, participants still felt ashamed at their imperfections.

#### Overarching theme: Relationship between AN, shame and perfectionism

##### Seeking pride

Participants described a cyclical relationship between shame and perfectionism that drove their descent into AN. For all participants, AN became a source of pride, which functioned to suppress shame (albeit temporarily). Participants spoke about the sense of accomplishment in being able to restrict their eating and set targets that they were able to meet. This was different from striving for the perfect body (as discussed above), as pride in dietary restriction resulted from being able to restrict food intake more than other people.

it does give you a sense of accomplishment if you make a plan, I'm only going to eat this today, and then you achieve that, it does make you feel, it makes you feel like you have achieved something, even if you have achieved nothing else in the day. Participant 4

Participants also spoke about feeling inferior to others in almost every way; however, one area they felt they could compete with others was with through attaining a certain kind of body. They felt they were not as successful as others or worthy as others but they felt they could be as thin as others.

I always felt inferior, and then, you know, I started losing weight, and that was the first time I ever felt, not better, but I felt like I didn't mind comparing myself to others. Participant 1

In the early stages of AN, many participants described being complimented on their weight loss and this gave them a sense of pride. They felt being perfect at AN was a way to alleviate their shame.

I'd lost like a little bit of weight and everyone was saying, like oh you look really good, like you look amazing, erm so it just spurred me on even more. Participant 5

This drive for thinness also interplayed with a sense of wanting to be the ‘perfect anorexic’, as often this was the only thing that they had in their lives that they felt that they could be perfect at.

Well, now I'm committed, I've got to stick with it or now I've got to do my absolute best in it. Participant 3

##### Numbing shame

Participants often spoke about how they did not know how to deal with emotional distress, often stemming from their past.

When you have a headache, you know, you hold your head. When you have a stomach ache, you hold your stomach. But when you feel sad, like, what do you hold, what do you do? Participant 9

However, by restricting their eating, they were able to convert this emotional pain into physical pain which they felt was easier to manage.

Participants spoke about AN generally numbing all emotions including shame.

I think a big part of it is that if I engage in the anorexic behaviours, if I'm restricting, my emotions are numb, so I don't have to feel those emotions, it just numbs me from everything. Participant 5

They described this as a positive thing as it meant they did not have to deal with their negative emotions and feeling nothing was better than feeling shame. Some participants noted that the AN only gave a temporary relief from shame and when the feelings came back they came back stronger.

##### Shame at breaking dietary rules

After people had developed AN and were trying to restrict their food intake, the AN would feed back into their shame and they would experience intense shame at breaking dietary rules or feeling as if they had overeaten. Not being able to stick to these dietary rules affected people's sense of self‐worth and created a significant amount of shame:

If I have a biscuit it doesn't just mean that I have messed up my diet, it means that I have messed up everything in my life. Participant 8

##### Shame at having anorexia

Participants also expressed AN feeding back into shame by being shameful itself, and thus intensifying their feelings of shame. All participants expressed that at some time they had been ashamed of the fact they had AN.

obviously I'm ashamed that as a 41 year old woman, that I can't pick up a knife and fork and eat a sensible meal. Participant 2

Participants also expressed shame at all of the things they had missed out on because their life had been dominated by AN. They spoke about comparing themselves to their peers and thinking about what their lives could have been like if they did not have AN. They spoke directly about missing out on relationships, their career, having children, etc. This related to their perfectionism as they felt shame at not having a perfect life.

I think looking back, I see how much of my life has been overshadowed by the eating disorder, and yeah I am ashamed, I am ashamed of who I am and who I have become, who I have missed out on being and all of these opportunities that I missed out on… Maybe if I hadn't had the anorexia I would have met someone by now, a lot of my relationships have suffered because of the anorexia, I can't physically have children because I don't have periods, that is because of the anorexia. Participant 5

#### Overarching theme: Recovery

All participants spoke about the conflict between wanting to recover and wanting to continue with the AN. Participants were at different stages of contemplating recovery and also expressed that this motivation for change fluctuates.

It can feel like I'm caught between a rock and a hard place with that. I mean, on one hand I might feel shame because I'm maybe not doing as good as I can with my recovery, but at the same time, you know, if I'm not feeling shame about that, I'll be feeling shame about not following my anorexic thoughts or not doing the most I could with the anorexia, kind of failing as an anorexic. Participant 8

##### Who am I?

A key feature of this struggle in recovery was how AN was perceived as being a central part of their identity and when they contemplated recovery, participants did not know who they would be without the AN. There was a sense that participants would be ‘nothing’ without AN and this linked back into their shame. The fact that they could be ‘perfect’ at AN alleviated their shame and impacted on their sense of self, so participants were reluctant to give that up.

Yes, it is frightening. Yes. You take them away. And then, you know, what if I am left with nothing? It's like jumping off a cliff. You don't know where you are going to land, or anything. Participant 11

This analogy about recovery being like jumping off a cliff was shared with another participant, but this participant was ready to jump;

Like it's just the unknown I think. But I'd rather jump in to the unknown than carry on living with something that I know is not a nice way to be living. Participant 8

##### Perfect recovery

Interestingly, perfectionism had one final twist in the participant's story. A number spoke about the need for a ‘perfect recovery’ and this became a large barrier to recovery as people did not want to try until they could ensure they could fully commit to it. Participants felt that to try and not succeed meant they were a failure and this would evoke more shame, but if they did not try they could not fail and thus would avoid the feelings of shame.

Your intention is to recover, like I say you can become obsessed with perfectly getting better, and if you don't succeed, then you can beat yourself up. Participant 7

## DISCUSSION

This is the first study to examine the relationship between perfectionism and shame in AN and how these constructs relate to recovery. In keeping with recent publications, this study found that shame and perfection in AN are linked to early trauma/aversive early experiences (e.g. Henderson et al., 2019). The findings suggested that early aversive experiences play a crucial role in the development of ‘core shame’ (Greenberg, 2006) where the self is seen as defective in some way, whilst perfectionism drives behaviour to manage these feelings of shame. As discussed by Skårderud (2007), these early experiences potentially lead to the development of global shame where the self is seen as damaged or flawed in some way. One potential hypothesis is that this global shame leads to very specific, focal shame that centres on poor body image. It is not surprising, perhaps, that this leads to a focus on trying to develop the ‘perfect’ body and the subsequent feelings of pride when this is perceived to have been achieved. As discussed by Fairburn et al. (2003), perfectionism is a crucial factor in eating disorders, and it is a core concept with their transdiagnostic model and CBT‐E (Fairburn, 2008). The findings of this study offer further support to the compassion‐focused therapy model of eating disorders (CFT‐ED; Goss & Allan, 2012) by highlighting the key role that shame plays within eating disorders. This model discusses how searching for pride, especially body pride, is used as an antidote to shame, but that it inadvertently reinforces the feeling of failure, when aspiring levels of pride are not achieved. This theoretical view was given more empirical support by Faija et al. (2017) who provided a more nuanced understanding of pride in AN, including different subtypes of pride. However, this study's findings highlight that pride needs to be considered in a more holistic way, as it is likely that pride could be regarded as a feature of perfectionism, as described by Fairburn (2008). A ‘vicious cycle’ relationship between shame and perfectionism was found in this study, the results also highlighted a number of other pathways from shame to AN, in which AN‐related thoughts and behaviours appeared to act as a powerful way to numb the unbearable feeling of shame. This is consistent with research by Harrison et al. (2011) who found that people with AN often have poor emotional regulation and lack a repertoire of strategies to manage emotions.

With regards to recovery, this study found that both shame and perfectionism had an impact on recovery. Shame influenced recovery in two ways. First, people were unsure of their identity and who they would be without AN, and went as far as questioning whether they would be nothing without AN. This is especially true when the finding regarding global shame is considered. A cross cutting theme from the results highlighted how participants felt flawed in some way and recovery is not likely to occur unless this core shame is addressed. This point is discussed by Allan and Goss (2012). Furthermore, as previous research has identified, people can feel they have become defined by their AN (Granek, 2007; Williams et al., 2016), and participants spoke about safety in familiarity and that recovery was a jump into the unknown, with some people feeling ready to jump and some not. Although a tentative hypothesis, it is likely that if someone still feels global shame and, thus, feels that they are nothing or flawed without AN, their recovery from their eating disorder will be compromised.

This is consistent with previous studies which identified that Second, as discussed above, participants were unsure and frightened about how they would deal with these difficult and painful emotions meaning that their motivation to give up their AN was often quite low. Interestingly, perfectionism also had a profound influence on recovery as participants often felt trapped by the need to make a ‘perfect recovery’. This led to them being reluctant to move towards recovery out of fear that they would do it imperfectly.

### Limitations and strengths

Participants were all self‐selecting, meaning that only those that were able to recognise and express shame and perfectionism would take part in this study. In keeping with Faija et al. (2017), this sample would have had received psychological therapies which could have meant that they were socialised into some of the concepts discussed within the research study. However, this is unlikely to have had a big influence as many of the concepts under discussion are not necessarily covered as standard by conventional treatments (e.g. shame and the sense of self). There were a number of strengths in this study, such as participants drawn from clinical services meaning that there are more representative of the clients that clinicians would see in their everyday work. Further, there was a spread of ages, length of diagnosis and service experience meaning that the analysis could be more nuanced in considering the impact of shame and perfectionism across all of the analysis. It is important to say that this study only recruited female participants and, therefore, these findings can only be applied to females. It would be important to repeat this study with males and/or people from other cultures/backgrounds with AN to see if there are similar patterns/processes.

### Research and clinical implications

The findings from this study highlight the importance of perfectionism and shame in the aetiology and maintenance of AN. The findings of this study highlight some key points, such as the role of early life/trauma in developing global shame and also how this relates to other phenomena within AN, such as the AN voice (e.g. Morrison et al. (2022)). Further work is being undertaken that is investigating the interaction between global shame and self‐disgust within body image.

This study also demonstrates how shame and perfectionism can be barriers to recovery. Treatments should focus on targeting both perfectionism and shame to successfully treat AN, and compassion focused therapy (CFT) in eating disorders to increase self‐compassion and reduce shame has had promising results (Gale et al., 2014; Goss & Allan, 2014).

The study's findings in terms of the extent of shame's importance in driving perfectionism in AN, and perhaps in some cases being a precursor to perfectionism, suggest that a re‐examination of traditional treatments to include shame is warranted. CBT‐E already includes a module on perfectionism when it is a strong feature in the patient's maintenance formulation and in particular where it is directed towards behaviours additional to the eating disorder (Fairburn, 2008); however, an examination of shame – particularly in those who report traumatic early life experiences that may have contributed to it – may be particularly relevant for those patients who have not recovered from first‐line treatments, either as an additional module or as part of a treatment that already prioritises a focus on shame (e.g. compassion focused therapy for eating disorders; Goss & Allan, 2012).

## AUTHOR CONTRIBUTIONS

**Tina L. M. Howard:** Conceptualization; data curation; formal analysis; investigation; methodology; writing – original draft; writing – review and editing. **Marc O. Williams:** Conceptualization; data curation; formal analysis; investigation; methodology; writing – original draft; writing – review and editing. **Debbie Woodward:** Conceptualization; data curation; project administration; supervision. **John R. E. Fox:** Conceptualization; data curation; formal analysis; investigation; methodology; project administration; supervision; writing – original draft; writing – review and editing.

## CONFLICT OF INTEREST

All authors declare no conflict of interest.

## Data Availability

Data is not available from this study.
